# Acute Myocarditis Following Enteropathogenic Escherichia coli Gastroenteritis in a 30-Year-Old Male

**DOI:** 10.7759/cureus.78961

**Published:** 2025-02-13

**Authors:** Natalie B Saliba, Kenneth J Adams, Karilyn Sims, Emily Fontan, Joshua C Hollingsworth

**Affiliations:** 1 Medical School, Edward Via College of Osteopathic Medicine, Auburn, USA; 2 Pharmacology, Edward Via College of Osteopathic Medicine, Auburn, USA

**Keywords:** bacterial myocarditis, enteropathogenic e. coli, gastroenteritis complication, internal medicine-cardiology, internal medicine specialist

## Abstract

A 30-year-old male with a medical history of hypertension, severe obesity, and sleep apnea presented with enteropathogenic *Escherichia coli *(EPEC) gastroenteritis and was admitted to the hospital with no abnormalities noted on cardiac examination. On the third day of admission, the patient reported chest pain with an EKG revealing ST-segment elevations in the inferior leads and cardiac troponins reaching 4435 ng/L. The diagnosis of EPEC gastroenteritis and associated myocarditis was made through clinical evaluation and laboratory findings. The patient was discharged from the hospital on metoprolol and in stable condition.

EPEC is a rare cause of myocarditis, making the association between the two challenging to identify. Therefore, acute myocarditis should be considered in young patients presenting with acute coronary syndrome (ACS) symptoms in the absence of ACS risk factors. By identifying the association between gastroenteritis and myocarditis, this case emphasizes the importance of early diagnosis to facilitate timely treatment and prevent complications.

## Introduction

Myocarditis is the nonischemic inflammation of the myocardium that is caused by infectious agents, toxins, or immune-mediated conditions [1]. Viruses are among the leading infectious causes of myocarditis, with coxsackievirus B, parvovirus B19, and adenovirus being the most frequently implicated pathogens. Less common infectious causes include the bacterium *Borrelia burgdorferi,* associated with Lyme disease, and the protozoan *Trypanosoma cruzi*, the causative agent of Chagas disease [1]. *Escherichia coli*-induced myocarditis is especially rare with eight reports made from 1980 to 2019 in PubMed [2]. *Escherichia coli*-induced myocarditis has been confirmed only twice in previous cases through histopathology, revealing an accumulation of neutrophils in the myocardium and abscesses at the atrioventricular node [2].

The usual clinical presentation of myocarditis is a prodrome of fever, myalgia, and respiratory symptoms or gastroenteritis, followed by cardiac symptoms ranging in severity from electrocardiogram (EKG) abnormalities mimicking ST-elevation myocardial infarction (STEMI) to hemodynamic collapse [3]. The reported incident rate of viral myocarditis is 10 to 22 per 100,000 individuals, with bacterial myocarditis being even rarer [4]. However, the true incidence of myocarditis is unknown due to the infrequent use of endomyocardial biopsy [1]. Many affected by myocarditis are initially asymptomatic and later diagnosed with heart failure due to cardiomyopathy [3]. The most common complication of myocarditis is nonischemic dilated cardiomyopathy, with other common complications including sudden death, arrhythmias, complete heart block, and acute myocardial infarction-like syndrome [1].

Challenges to diagnosing acute myocarditis include the non-specific symptoms that overlap with other conditions, such as fever, fatigue, and general discomfort associated with gastroenteritis. Another challenge to diagnosing acute myocarditis is the presence of a normal initial workup, including chest radiographs and EKGs. With chest pain only presenting in approximately 32% of cases, it remains difficult to recognize the signs of myocarditis that could be attributed to other conditions [4]. A recent study recommends considering acute myocarditis in young patients presenting with acute coronary syndrome, particularly when traditional, non-modifiable coronary risk factors are absent, EKG abnormalities involve multiple coronary arteries, or echocardiography reveals global rather than segmental left ventricular dysfunction [5].

We present a case illustrating a potentially life-threatening connection between enteropathogenic *Escherichia coli* (EPEC) gastroenteritis and the subsequent development of myocarditis.

## Case presentation

A 30-year-old male with a medical history of hypertension, severe obesity, and sleep apnea presented to the emergency department with complaints of fever, nausea, and vomiting. The patient consumed undercooked pork the night prior and within six hours, started vomiting without hematemesis. Upon arrival at the emergency department, the patient was febrile at 101.5 °F with sharp abdominal pain, a blood pressure of 121/48 mmHg, a heart rate of 112 beats per minute, respiratory rate of 16 breaths per minute, and an O2 saturation of 99% while breathing room air. The physical examination was within normal limits, with an emphasis on no abnormalities in cardiopulmonary or abdominal examination. The patient denied chest pain, shortness of breath, and diarrhea. The patient denied the use of alcohol, tobacco, and illicit drugs.

**Figure 1:**
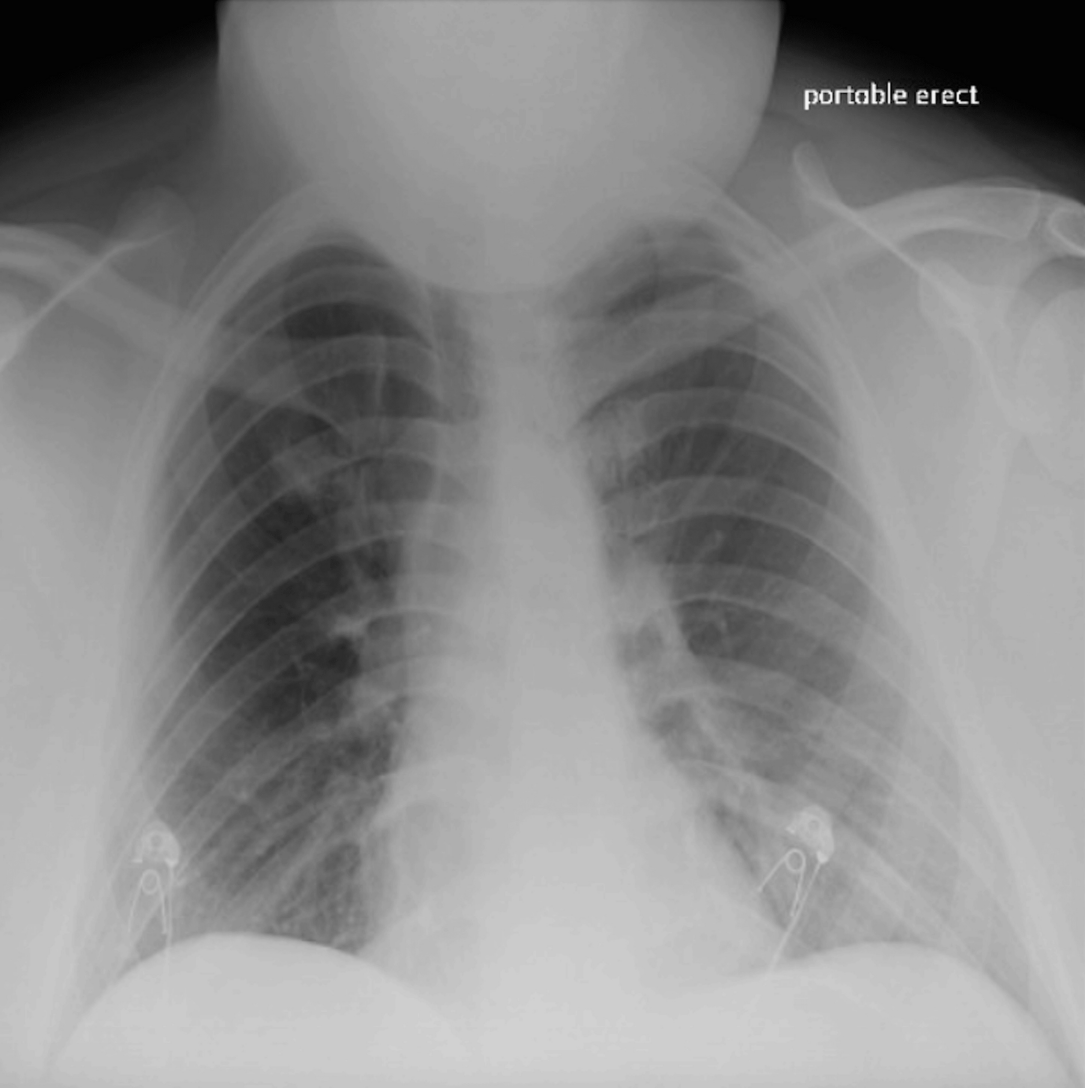
The chest radiograph is unremarkable, with no significant findings.

The initial EKG revealed sinus tachycardia. Chest radiography did not reveal any acute pulmonary processes and noted normal heart size (Figure 1). Laboratory results revealed leukocytosis at 12.3 cells/µL. The patient was diagnosed with gastroenteritis and sepsis. He was admitted and started on intravenous fluids with an intravenous broad-coverage antibiotic, cefepime, while awaiting blood cultures and a gastrointestinal panel.

**Figure 2:**
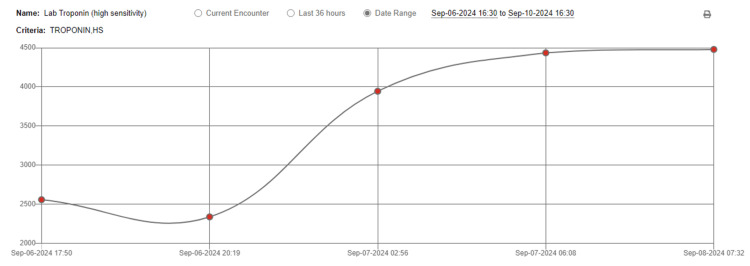
Cardiac troponins were 2767 ng/L on the third day, plateauing at 4435 ng/L on the fifth day.

On the second day, the patient’s temperature was 102.5 °F, blood pressure 100/48 mmHg, heart rate of 110 beats per minute, and respiratory rate of 20 breaths per minute. Leukocytes decreased to 9.0 cells/µL within the normal range. The gastrointestinal panel revealed EPEC, leading to the continuation of cefepime with the plan to transition to levofloxacin upon discharge. Over the course of the third and fourth days following admission, the patient’s vitals and leukocytes were within normal limits, but the patient reported on the fourth day having worsening chest pain for the last 24 hours. An EKG was performed, revealing sinus tachycardia and 0.5 mm elevation of the ST-segment in the inferior leads. Cardiac troponins were 2767 ng/L, plateauing at 4435 ng/L on the fifth day (Figure 2). Based on EKG abnormalities, a clinical diagnosis of myocarditis was suspected, leading to additional testing to exclude acute coronary syndrome as a diagnosis.

On the fifth day, an echocardiogram revealed a normal ejection fraction (EF) of 55%. Trace mitral and trace tricuspid regurgitation were noted with normal pulmonary artery pressure and no pericardial effusion. On the sixth day, cardiac catheterization was performed, showing no significant coronary artery disease, an elevated left ventricular end-diastolic pressure (LVEDP) of 27 mmHg, and normal left ventricular systolic function with an estimated EF of 55% with normal wall motion. Following this, the patient was discharged with discontinuation of heparin and aspirin. Metoprolol 12.5 mg was started due to LVEDP elevations with instructions to follow up with a cardiologist. The patient was asymptomatic at the time of discharge and reported feeling improvement.

## Discussion

EPEC gastroenteritis may occur when food or water that is consumed is contaminated with feces due to improper sanitation. The typical onset is within four to 12 hours with symptoms of watery diarrhea, fever, and vomiting [6]. *Escherichia coli*-induced myocarditis is rare and usually associated with other risk factors such as advanced age, chronic comorbid conditions, or dissemination through a urinary tract infection [2].

The exact pathogenesis of EPEC-myocarditis is unknown, but two proposals have been made. One proposed mechanism involves an outer-membrane protein only present in gram-negative bacteria, called the peptidoglycan-associated lipoprotein (PAL). Mouse models have shown that the PAL is released in the bloodstream during *E. coli* sepsis, infiltrating receptors on cardiomyocytes. This further causes dysfunction and inflammation of the myocardium through tumor necrosis factor (TNF)-α [7]. A second mechanism proposes that there is a lipopolysaccharide-induced production of TNF-α, interleukin-6 and nitric oxide that causes inflammation and injury to the myocardium [8]. Other theories have suspected that the myocarditis does not result from the EPEC infection itself, but is triggered by an inflammatory or immune response secondary to infection. This could explain the pathogenesis if myocarditis develops weeks after the initial infection, as immune-mediated responses often require time to fully manifest during an infection [9].

A definitive diagnosis of myocarditis can only be made with an endomyocardial biopsy (EMB), which is rare due to its invasiveness and is only recommended in particular cases. The clinical diagnosis is usually done through a diagnosis of exclusion. However, myocarditis is difficult to distinguish from acute coronary syndrome, pericarditis, and valvular disease [4]. An echocardiogram can distinguish between acute myocarditis and chronic myocarditis which usually presents with an increased thickness of the left ventricular wall due to myocyte loss, fibrosis, and the progression to dilated cardiomyopathy. Cardiac catheterization can be performed on patients in an effort to distinguish acute myocarditis from acute coronary syndrome (ACS) and atherosclerosis. Cardiac catheterization can be used to measure LVEDP. Similar to our case, an elevated LVEDP indicates impaired ventricular relaxation, increased stiffness, or volume overload, which are common in myocarditis. However, longstanding obesity or hypertension can also contribute to these findings, making it challenging to determine the primary cause in this context. The presence of cardiac enzymes is a marker of inflammation or injury which can be seen in ACS or pericarditis. In acute myocarditis, approximately 92.6% of patients present with EKG findings such as ST-segment changes, which are not present in pericarditis [4]. With the combination of these tests, the clinical diagnosis of myocarditis can be made on the basis of exclusion and an invasive endomyocardial biopsy can be avoided. If the patient lacks improvement, endomyocardial biopsy may be warranted to determine the need for immunosuppressive therapy and prognosis [10]. The plan of treatment of myocarditis in improving patients with other cardiac possibilities excluded remains the same, regardless of definitive diagnosis.

The treatment of myocarditis is supportive, with an emphasis on bed rest, continuous monitoring, and treatment of the underlying cause with antibiotics [11]. If complications arise, management is dependent on the complication. For example, external pacing is required in the presence of an arrhythmia [11]. First-line medications for the recovery phase of myocarditis to protect the myocardium are angiotensin-converting enzyme (ACE) inhibitors or angiotensin receptor blockers. Negative inotropic agents, such as metoprolol, are used with caution in the recovery phase to prevent further damage and progression to chronic myocarditis. Beta-blockers are known to reduce the workload of the heart to prevent further damage, with risks of hypotension, delayed recovery, or exacerbating heart failure, if present. In a 2009 study, metoprolol was noted to improve survival, cardiac remodeling, fibrosis, and left ventricular function [12]. In cases of comorbidities such as hypertension and severe obesity, metoprolol’s benefits can outweigh the risks to prevent the progression to chronic myocarditis or dilated cardiomyopathy. In healthy patients presenting with myocarditis, a complete recovery is expected over a few months if appropriate care is utilized [4].

## Conclusions

In conclusion, the management of this patient with *E. coli*-induced myocarditis stresses the importance for close monitoring of sepsis patients for potential cardiac complications. Early recognition of myocarditis remains difficult, especially when initial evaluations show no signs of cardiac involvement. However, this case highlights the importance of remaining vigilant in patients presenting with a bacterial or viral-like prodrome who develop new symptoms such as shortness of breath or chest pain. A thorough cardiac workup, even in the absence of obvious cardiac findings, is essential to ensure timely diagnosis and treatment. By recognizing subtle signs of myocarditis early, the patient can have a significantly different outcome, especially in critically ill individuals. This case serves as a reminder of the interplay between infectious diseases and cardiovascular health, emphasizing the need for a multidisciplinary approach in managing such patients. Ultimately, vigilance and a comprehensive evaluation are key to avoiding missed diagnoses and improving patient care.
